# Feature selection and classifier performance on diverse bio- logical datasets

**DOI:** 10.1186/1471-2105-15-S13-S4

**Published:** 2014-11-13

**Authors:** Edward Hemphill, James Lindsay, Chih Lee, Ion I Măndoiu, Craig E Nelson

**Affiliations:** 1 Department of Molecular and Cell Biology,Storrs, CT, USA; 2 Department of Computer Science and Engineering, Storrs, CT, USA

## Abstract

**Background:**

There is an ever-expanding range of technologies that generate very large numbers of biomarkers for research and clinical applications. Choosing the most informative biomarkers from a high-dimensional data set, combined with identifying the most reliable and accurate classification algorithms to use with that biomarker set, can be a daunting task. Existing surveys of feature selection and classification algorithms typically focus on a single data type, such as gene expression microarrays, and rarely explore the model's performance across multiple biological data types.

**Results:**

This paper presents the results of a large scale empirical study whereby a large number of popular feature selection and classification algorithms are used to identify the tissue of origin for the NCI-60 cancer cell lines. A computational pipeline was implemented to maximize predictive accuracy of all models at all parameters on five different data types available for the NCI-60 cell lines. A validation experiment was conducted using external data in order to demonstrate robustness.

**Conclusions:**

As expected, the data type and number of biomarkers have a significant effect on the performance of the predictive models. Although no model or data type uniformly outperforms the others across the entire range of tested numbers of markers, several clear trends are visible. At low numbers of biomarkers gene and protein expression data types are able to differentiate between cancer cell lines significantly better than the other three data types, namely SNP, array comparative genome hybridization (aCGH), and microRNA data.

Interestingly, as the number of selected biomarkers increases best performing classifiers based on SNP data match or slightly outperform those based on gene and protein expression, while those based on aCGH and microRNA data continue to perform the worst. It is observed that one class of feature selection and classifier are consistently top performers across data types and number of markers, suggesting that well performing feature-selection/classifier pairings are likely to be robust in biological classification problems regardless of the data type used in the analysis.

## Background

Due to the recent rise of big-data in biology, predictive models based on small panels of biomarkers are becoming increasingly important in clinical, translational and basic biomedical research. In clinical applications such predictive models are increasingly being used for diagnosis [1], patient stratification [2], prognosis [3], and treatment response, among others.

Many types of biological data can be used to identify informative biomarker panels. Common ones include microarray based gene expression, microRNA, genomic copy number, and SNP data, but the rise of new technologies including high-throughput transcriptome sequencing (RNA-Seq) and mass spectrometry will continue to increase the diversity of biomarker types readily available for biomarker mining.

Useful predictive models are typically restricted to use a small number of biomarkers that can be cost-effectively assayed in the lab [4]. The use of few biomarkers also reduces the effects of over-fitting, particularly for limited amounts of training data [5]. Once training data has been collected and appropriate procedures for normalization of primary data have been defined, assembling a robust biomarker panel requires the solution of two main computational problems: *feature selection*, to identify a short list of informative biomarkers, and *classification*, used to make predictions for new samples based on patterns extracted from the training data. Both of these steps have been explored extensively in the statistics and machine learning literature, and many alternative algorithms are available for each. Due to the sheer number of available choices and the lack of predictable interactions between feature selection method, classification algorithm, and data type, assembling the most robust biomarker assay for a given biomedical application is rarely undertaken systematically. Rather, it is more often driven by the intuition and a priori preferences of the statistician.

Available feature selection methods can be grouped into three broad categories: filter, wrapper and embedded. Filtering approaches use an easy to calculate metric which allows quick ranking of the features, with top ranking features being selected. Wrapper methods use a classification algorithm to interrogate the effect of various biomarker subsets. Embedded approaches are classification algorithms which eliminate features as part of the training process. Recent studies [6-8] investigated the influence of feature selection algorithms on the performance of predictive models and provided a framework for thorough comparison of approaches. However the effect of the number of biomarkers selected and high-dimensional data type was not explored.

There are hundreds of publications describing classification algorithms and their applications to genetic research and medicine. Many publications advocating a new method employ a limited comparison between similar approaches. However non-uniform validation strategies make it difficult to assess performance of a wide variety of approaches. A previous study compared both classification and feature selection approaches in a unified framework [8], however the effect of biological data type was not explored, but it was observed that the biological question does have an effect on the best model. Additionally most comparisons typically overlook the effect of model parameterization even though the choice of parameters can have profound effects on performance.

This work presents a large scale empirical comparison of the effects of the interaction between the main components of the predictive model (i.e., feature selection and classification algorithms), the number of features utilized, and the underlying data type on the performance of the overall model. This study also implements exhaustive parametrization of all models to ensure a fair comparison between models.

In order to test the performance of the large number of models tested in this study, and in order to be able to run direct comparisons of the models on different biological data types, we took advantage of the publicly available NCI-60 cancer cell line data set [9]. The NCI-60 cell line collection represents a carefully curated collection of 60 independent cancer cell lines derived from nine types of cancer occurring in 60 individual patients. Each line has been uniformly cultured and DNA fingerprinted to ensure independence [10]. In addition, the NCI-60 cell lines have been subjected to extensive molecular characterization including mRNA microarray [11], microRNA [12], protein lysate arrays [11], SNP arrays [13], and aCGH analysis [14]. For these reasons, the NCI-60 data set represents a tremendous research tool for exploring and benchmarking Omics-type approaches to cancer classification and therapeutics.

Cancers are widely believed to derive from a single event in which one cell escapes the many surveillance mechanisms in place to prevent uncontrolled proliferation. Once this has occurred, the cancer often evolves quickly, rapidly acquiring large numbers of mutations, ranging from small point mutations to very large chromosomal aberrations and regional amplifications (DNA duplications). The original identity of the cancer cell (its cell type or tissue type) appears to exert a very strong influence on the course of evolution of the cancer. For this reason, characteristic mutations will often be found in cancers derived from the same tissue, even in different patients. In addition, because identical cell types from different patients will share very similar gene expression signatures, cancers derived from these tissues will often do the same. In the present study we take advantage of these two features of cancer to test the ability of various statistical models to correctly infer the cell type (or "tissue-of-origin") of each cancer cell line. The ability to make this inference correctly not only represents an excellent test of these models on real biological data, it is a good example of the type of classification ability required for targeted cancer therapeutics.

## Methods

### NCI-60 cancer cell-line dataset

In order to test the predictive models in this study we use publicly available data from the NCI-60 cancer cell lines as provided by CellMiner [9]. For the purpose of this study, we analyzed cancers with at least 5 representative cell lines derived from the same tissue-of-origin (5-9 cell lines per tissue-of-origin). These lines represent cancers emerging from eight tissues: breast, central nervous system, colon, leukemia, melanoma, non-small cell lung, ovarian, and renal cancers. The data types used in this study are gene expression (mRNA) and protein lysate (protein) arrays [11], microRNA [12], SNP arrays [13], and array comparative genome hybridization (aCGH) [14]. All data has been normalized according to best practices for each assay platform prior to downloading for this study [9]. The specific cell lines and data files used in this study can be found in Supplemental Tables S1 and S2.

### Feature selection methods

The area of feature selection in machine learning has recently been quite robust. There are numerous specialized feature selection algorithms which identify the most informative biomarkers from high-dimensional data. This study utilized at least one approach from each of the three broad categories identified above (filter, wrapper, and embedded). Every approach utilized allowed for a specific number of features to be chosen. No requirement was established that induced a relationship between feature sets from the same algorithm. So the 16 features chosen by one approach are not required to be a subset of the 32 features chosen by the same. For all algorithms we used the implementations in the Scikit-learn [15] Python package, please refer to its associated documentation for specific implementation details.

The fastest and most simplistic selection method is univariate filtering. These approaches rank features according to some score, and the user selects the best k features accordingly. Here the F-statistic (Anova), a generalization of the t-test, is used as a filter, as suggested in [8] and [6]. There are no parameters for this feature selection method.

Wrapper approaches typically use some type of greedy strategy to select influential features using a black box classifier. They are more computationally intensive, however SVM recursive feature elimination (SVM-RFE) is extensively used in medical applications [16]. The parameters considered were the penalty parameter and loss function.

The final class of feature selection algorithms is embedded approaches where the features are chosen while building the classifier. To represent this class two tree-based methods were adapted; random forest (RF) [17] and extra-trees (ET) [18]. The parameter considered was the number of trees used in each approach.

A summary of parameters of all considered feature selection methods along with the range of values searched for each parameter are given in Supplemental Table S3.

### Classification methods

An exhaustive comparison of all classification algorithms would be quite challenging. Therefore only a small number of approaches was explored, chosen to represent most common machine learning approaches used in bioinformatics. Identifying the cancer type from the NCI-60 dataset is inherently a multi-category classification problem. Therefore each considered approach must accommodate this setting or be adaptable by one-vs-one [19] or equivalent approaches. The types of algorithms tested fall into three main categories: linear, tree, and distance based methods. Again we used the Scikit-learn [15] Python implementations for all compared classification algorithms.

Linear classifiers use a linear function to score classes by taking the dot product of feature values and feature weights computed during training. One of the most powerful, flexible and ubiquitous linear classifier is the support vector machine (SVM) with linear kernel [20]. SVM has been utilized in numerous works describing predictive models with biological and medical significance. Both the penalty and loss function parameters were explored. Another powerful linear classifier is logistic regression (LR) [21]. The specific implementation uses one-vs-all to accommodate the multi-classification setting instead of the one-vs-one approach. The penalty function, and regularization parameters were explored.

Classification trees are a machine learning tool which has found extensive use in the biological and medical communities. This is partially due to both their resilience to over-fitting and ease of interpretation. This work looks at three related approaches; vanilla decision trees (DT) [22], random forest (RF) [18] and gradient boosting (GB) [23]. Decision trees represent class labels as leaves in the tree and branches are combinations of features that lead towards a leaf. Vanilla decision trees can often over-complicate the explanation necessary to arrive at the appropriate class label, however their interpretation is very simple. Random forest approach and gradient boosting are ensemble learning techniques where multiple trees are created and the final decision is some aggregate. These approaches are less-susceptible to over-fitting however they are often computationally intensive. The common parameter explored is the number of trees used and for gradient boosting the number of boosting stages.

Distance based methods surveyed are k-nearest neighbors (KNN), cosine (Cos) and correlation (Corr). Cosine and correlation are simple classifiers which calculate the distance to all training samples from the test sample and assigns the label based on the closest match. KNN is a slightly more advanced version of the same concept in which class membership is assigned by majority voting among the *k * closest matches.

A summary of parameters of all considered classification algorithms along with the range of values searched for each parameter are given in Supplemental Table S4.

### Validation strategy

**Figure 1:**
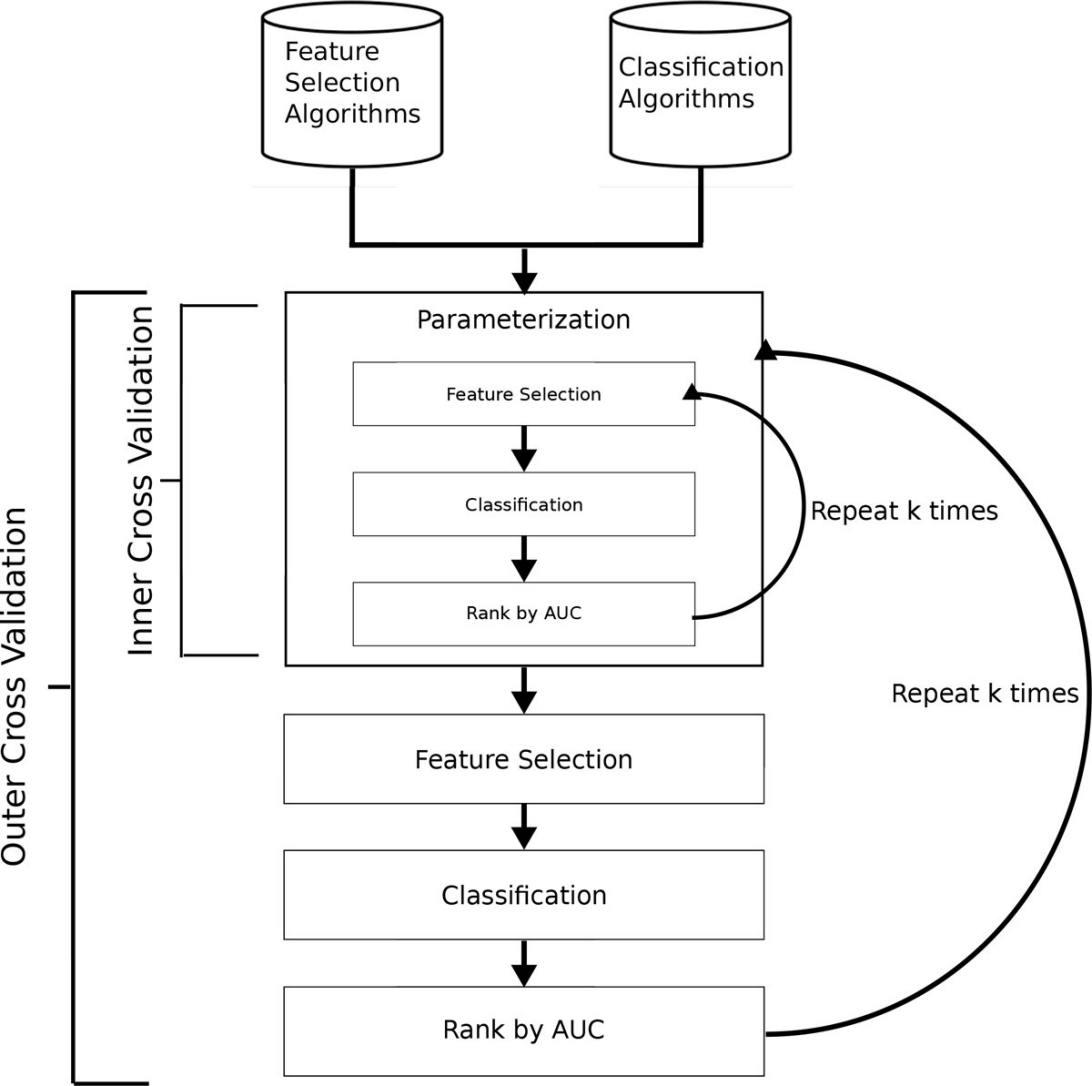
****Validation strategy****. Flow chart of the validation strategy. First all combinations of feature selection and classification algorithms (4***x***9) are parametrized in the inner ***k***-fold cross-validation loop based on the training folds of the outer ***k***-fold cross-validation. The best parameters are found by maximizing AUC. Once the parameters are fix the outer ***k***-fold cross-validation loop is run and the average AUC (or similar metric) is recorded.

A common validation strategy used in evaluating machine-learning methods is *k*-fold cross-validation [6,8]. Here the data is partitioned into *k *equal size subsets with each set used in turn for testing while the other *k − *1 subsets are used as training data. Care must be taken taken to avoid substantial biases [24] by ensuring that feature selection is performed only on the data reserved for training. Since the approach presented here is also parameterizing for each distinct model, nested *k*-fold cross-validation is used to tune parameter values. This requires an additional cross-validation experiment on each training dataset, where a grid-search over the considered parameter range is performed. The inner phase identifies the best parameter values which are then used exclusively in the outer cross-validation. In order to build stronger evidence for the models' performance, the outer cross-validation phase was repeated 100 times, however the parameterization was only performed in the first iteration. Biases towards selecting more complex models with more parameters or overly fine grid-steps are still a possibility, however nested cross-validation should largely mitigate them. More advanced techniques presented in [25] could be utilized in future iterations. An outline of the validation strategy can be seen in Figure 1.

The nested *k*-fold cross-validation strategy is computationally very intensive. With 4 *× *9 = 36 models (combinations of feature selection and classifier) to evaluate, dozens of parameter values and different number of selected markers there can be upwards of 1,000,000 individual classifier runs per data type. The majority of the jobs occur in the inner cross-validation loop, and fortunately can all be run in parallel on a cluster or multi-core server. Also, a pre-filtering heuristic was applied to speed up the feature selection process. For all datasets with more than 1,000 features we retained only the top 1,000 features as ranked by the F-statistic prior to any additional feature selection.

To further validate the results on external datasets, eight primary tumor cohorts from The Cancer Genome Atlas (TCGA) were identified to match five NCI-60 tissue-of-origin cell lines; central nervous system, colon, non-small cell lung, ovarian, and renal. The mapping of the TCGA cohorts to the NCI-60 cell lines can be found in Supplemental Table 7. The TCGA derived gene expression microarray data was obtained from the Broad Institute's GDAC Firehose utility [26-34]. The presented pipeline was used to select biomarkers, identify and train the most informative model using NCI-60 data [35]. Then its performance was tested using the TCGA derived data.

### Metrics

There are numerous metrics used in evaluating the accuracy of a predictive model. One common metric is AUC, or *area under the receiver operating characteristic (ROC) curve*. The ROC curve is a plot of the true positive rate against the false positive rate. The AUC is then the area under this curve and is used as a single measurement of classifier performance. This definition is typically for binary classification tasks, however there are several extensions to multiclass classification problems [36]. Since the classes are equally represented in the NCI-60 dataset this work utilizes the multiclass metric, $AUC_{total}=\sum_{c_{i}\in C}AUC\left(c_{i}\right)\cdot p\left(c_{i}\right),$ where *AUC*(*c_i_*) is the typical binary classification AUC for class *c_i _*and *p*(*c_i_*) is the prevalence in the data of class *c_i_*.

## Results and discussion

**Figure 2:**
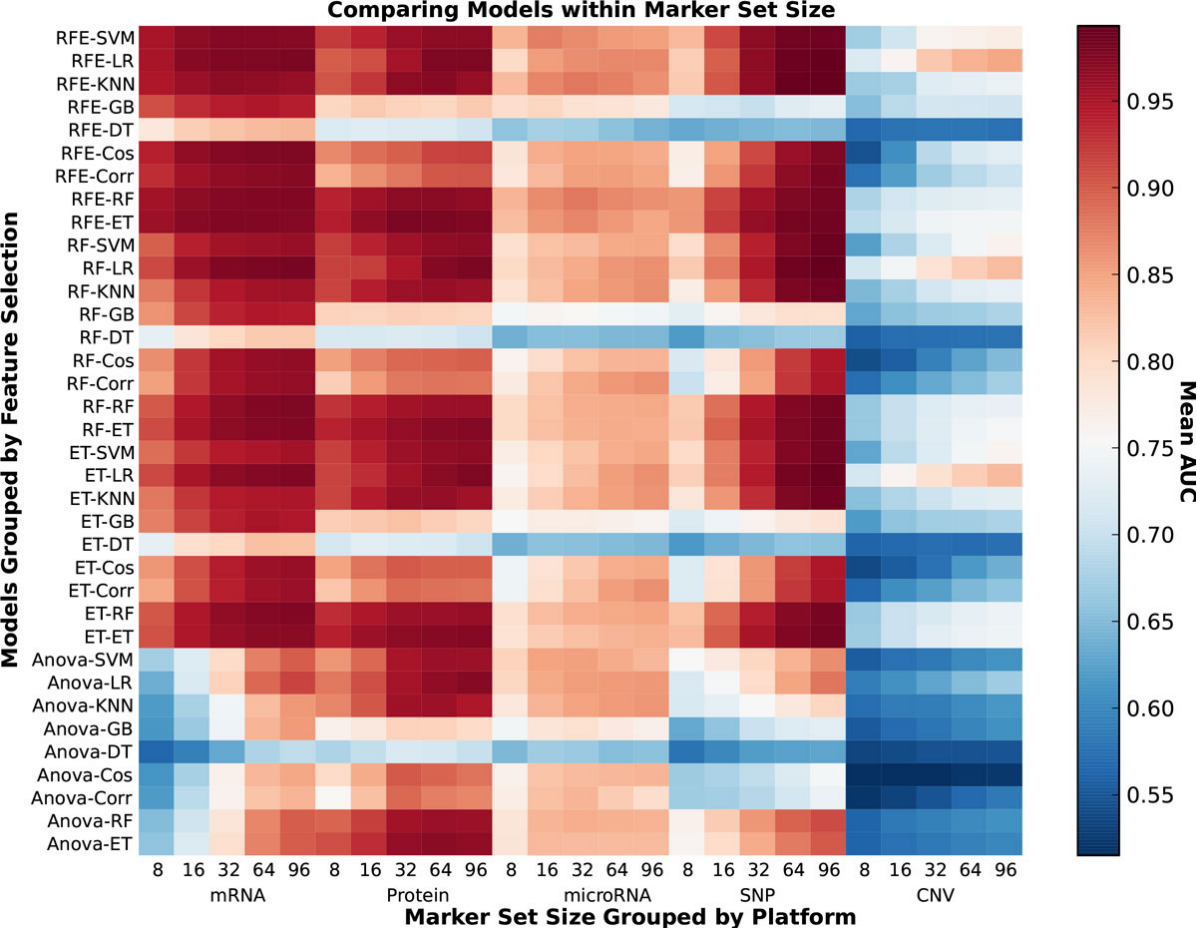
**AUC heatmap**. This heatmap contains the average AUC for each model (grouped by feature selection) for each data type at each number of markers. The darker the block, the more accurate the predictive model is.

This study is evaluating the effect of three parameters simultaneously: the model, the data type and the number of markers. Therefore conclusions about the best predictive model are presented from the perspective of each parameter individually. In Figure 2 an overview of the AUC for each model, data type and each number of markers is presented as a heatmap. The hotter entries represent higher AUC.

### Model effects

**Figure 3:**
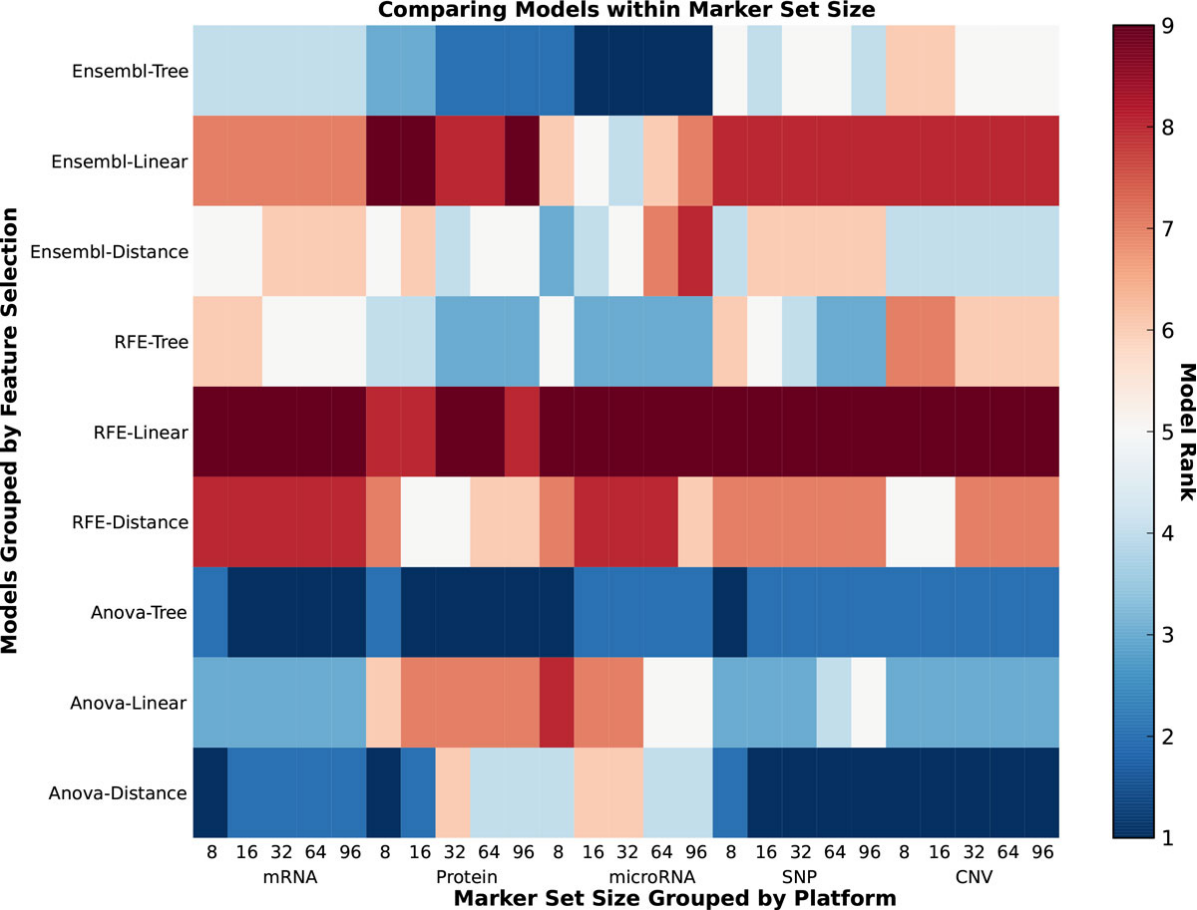
**Model rank**. This heatmap contains the relative rank based on AUC of each model across all data types. The darker spots indicate higher AUC and rank.

The accuracy of the predictive models varies greatly, with the various combinations of feature selection and classification algorithms. If the feature selection and classification algorithms are grouped by class, a high-level ranking becomes much clearer. In Figure 3 the relative ranking of each model is indicated by color for each data type at each number of features. The RFE-Linear combination which uses SVM-RFE for feature selection and logistic regression or SVM for classification is the best performing model in almost all instances. Close behind is Ensembl-Linear, where in Table 1 it is clear that it performs only slightly worse than RFE-Linear.

**Table 1:** AUC by data type and marker count.

Marker Set Size	SNP			mRNA			CNV			microRNA			Protein		
**8**	**RFE**	**ET**	**0.8598**	**RFE**	**ET**	**0.9585**	**RFE**	**LR**	**0.7198**	**RFE**	**RF**	**0.8352**	**RFE**	**ET**	**0.9426**
	RFE	RF	0.8591	RFE	RF	0.9554	ET	LR	0.7115	RFE	SVM	0.8352	ET	ET	0.9394
	RFE	SVM	0.8321	RFE	SVM	0.9521	RF	LR	0.71	RFE	KNN	0.8295	RFE	RF	0.9382
	ET	ET	0.8295	RFE	LR	0.951	RFE	ET	0.691	RFE	ET	0.8275	RF	ET	0.9376
				RFE	KNN	0.9467	RFE	RF	0.6802	Anova	SVM	0.8089	ET	RF	0.9312
										Anova	LR	0.8051	RF	RF	0.9272
										RF	ET	0.8028			
										RF	RF	0.8027			
										RFE	LR	0.8021			
										RF	LR	0.802			
**16**	**RFE**	**ET**	**0.922**	**RFE**	**ET**	**0.972**	**ET**	**LR**	**0.7616**	**RFE**	**SVM**	**0.8758**	**RFE**	**ET**	**0.9666**
	RFE	RF	0.9162	RFE	LR	0.9709	RFE	LR	0.7607	RFE	KNN	0.8704	ET	ET	0.9582
	RFE	SVM	0.9111	RFE	RF	0.9681	RF	LR	0.7468	RFE	RF	0.8671	RFE	RF	0.9565
	RFE	KNN	0.9033	RFE	SVM	0.968				RFE	ET	0.8597			
	ET	ET	0.8997	RFE	Cos	0.9663				RFE	LR	0.8535			
	RFE	LR	0.897							Anova	SVM	0.8496			
	RF	ET	0.896												
	ET	RF	0.8914												
**32**	**RFE**	**LR**	**0.9685**	**RFE**	**LR**	**0.9759**	**RFE**	**LR**	**0.8194**	**RFE**	**KNN**	**0.8806**	**RFE**	**ET**	**0.9792**
	RFE	SVM	0.9674	RFE	ET	0.9757				RFE	RF	0.8801			
	RFE	KNN	0.966	RF	LR	0.9747				RFE	ET	0.8717			
	RFE	ET	0.9646	RFE	Cos	0.9736				RFE	SVM	0.8679			
	RFE	RF	0.9577	RFE	RF	0.9734				RFE	LR	0.866			
				RFE	SVM	0.9734									
**64**	**RFE**	**KNN**	**0.9911**	**RF**	**LR**	**0.9789**	**RFE**	**LR**	**0.8379**	**RFE**	**KNN**	**0.8746**	**RFE**	**ET**	**0.979**
	RFE	LR	0.9892	RFE	LR	0.9777				RFE	LR	0.8688	RFE	LR	0.9782
	RF	LR	0.9862	RFE	Cos	0.977				RFE	RF	0.8682	RF	LR	0.9731
	RFE	SVM	0.9843	RFE	ET	0.976				RF	LR	0.8595	RFE	KNN	0.9727
	ET	LR	0.9837	RFE	RF	0.9757				RF	Corr	0.8585			
				RF	RF	0.9755				RFE	ET	0.8578			
				ET	LR	0.9741				RF	KNN	0.8574			
				RF	ET	0.9737				RFE	SVM	0.8568			
				RFE	SVM	0.9733				Anova	KNN	0.8564			
				ET	RF	0.9728				Anova	LR	0.8557			
				RFE	Corr	0.9709				ET	LR	0.8539			
										RFE	Corr	0.8537			
										ET	Corr	0.8536			
										ET	KNN	0.852			
										RFE	Cos	0.8492			
**96**	**RFE**	**KNN**	**0.9933**	**RF**	**LR**	**0.9808**	**RFE**	**LR**	**0.847**	**RFE**	**LR**	**0.8697**	**RF**	**LR**	**0.979**
	RF	LR	0.9918	RFE	LR	0.9787	ET	LR	0.8292	RF	KNN	0.8657	RFE	LR	0.9779
	RFE	LR	0.9916	RF	RF	0.9774				RF	LR	0.8643	ET	LR	0.9768
	ET	LR	0.9909	RFE	Cos	0.977				ET	LR	0.8634	RFE	ET	0.9765
				RFE	RF	0.9762				RFE	RF	0.8633	ET	ET	0.9734
				ET	LR	0.9761				RF	Corr	0.863	RF	ET	0.973
				ET	RF	0.9758				ET	Corr	0.8629			
				RF	ET	0.9746				RFE	KNN	0.8628			
				RFE	ET	0.9744				ET	KNN	0.8613			
										Anova	KNN	0.8596			
										Anova	LR	0.8573			
										RFE	SVM	0.853			
										ET	RF	0.8483			
										RFE	Corr	0.8477			
										RF	SVM	0.8474			

Table of AUC for top performing models for each data type and grouped by marker set size.

**Figure 4:**
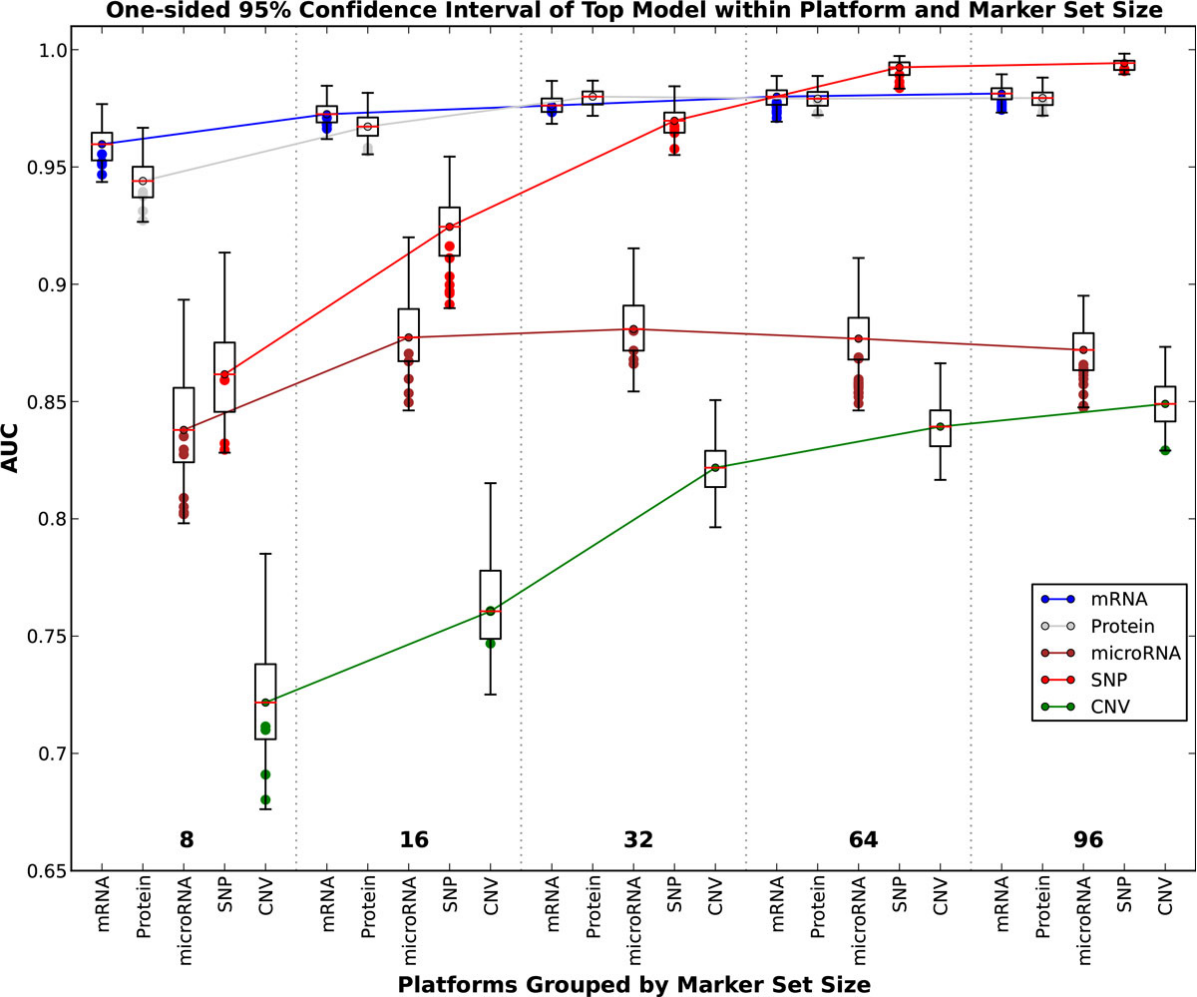
**AUC boxplots**. This figure contains box plots of the best model, for each data type and number of markers. The whiskers represent the 95% confidence interval, while the green dots represent another model with performance within the confidence interval.

**Figure 5:**
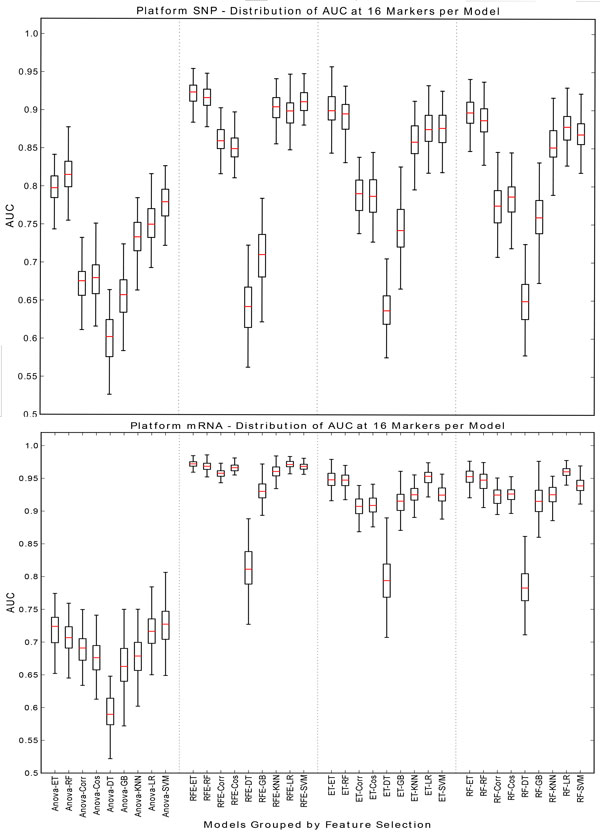
**SNP/mRNA: 16 markers**. This figure contains box plots describing the AUC of each model, grouped by the feature selection component for SNP and mRNA data type at 16 markers.

**Figure 6:**
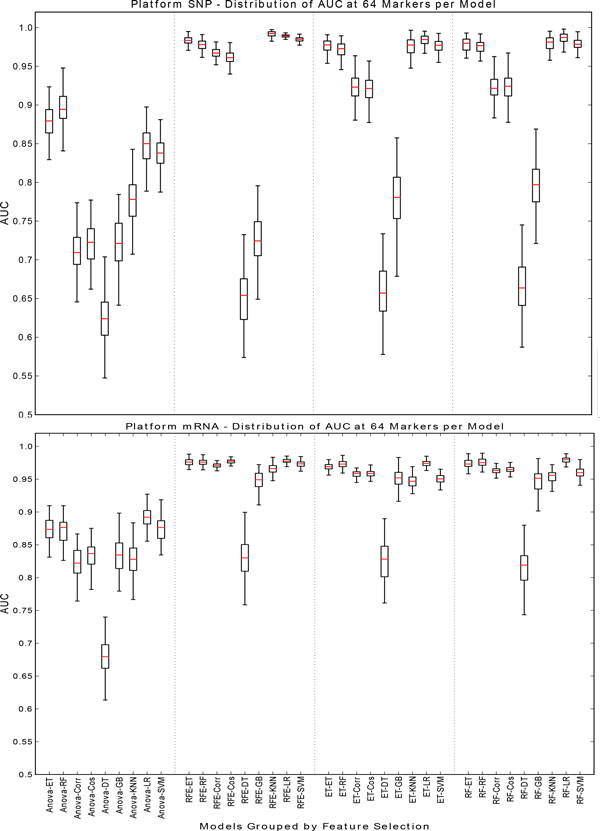
**SNP/mRNA: 64 markers**. This figure contains box plots describing the AUC of each model, grouped by the feature selection component for SNP and mRNA data type at 64 markers.

If the data type and number of features are fixed the effects of the models can be explored further. As seen in Figure 4 the mRNA and protein data types consistently afford the best classification accuracy at both high and low number of markers. Although classifiers have relatively poor performance on SNP data for 8 markers, as the number of selected biomarkers increases best performing classifiers based on SNP data match or slightly outperform those based on gene and protein expression. The accuracy of all models is generally highest at a high number of markers. Therefore mRNA and SNP at 16 (Figure 5) and 64 (Figure 6) markers were chosen to demonstrate model effects. Surprisingly, the effect of classifier choice is small as seen in Figure 3. The models are grouped by feature selection algorithm. For RFE there is very little difference between all the classifiers except decision trees and gradient boosting which are consistently poor performers. The major differences appear between feature selection groups, where SVM-RFE is the best, random forest and extra trees have equivalent performance, and Anova is the worst.

This conclusion is contrary to that of [6], where it was found that the t-test univariate filter (of which Anova is considered a multiclass generalization) often performed the best for feature selection. This could be due to the differences in the underlying complexity of the question; namely in [6] the goal was to predict metastatic relapse, which is a binary question, using gene expression microarrays. In addition, no parameter tuning using nested CV or similar approach was performed in [6]. Although this study cannot prove that a particular feature selection or classification algorithm is best in a certain scenario, it does indicate that a thorough model selection step is advised.

The relatively small effect of classifier choice is interesting and unexpected. This indicates that much more care should be given to choosing the right features, as this has the biggest effect on model performance.

### Data type effects

The rich selection of data types available for these cell lines provides the opportunity to compare the ability of many types of biological data to classify the tissue of origin of a tumor cell line. Some of these data types fundamentally reflect gene expression levels: mRNA, protein and microRNA. The other two: CNV and SNP, are generally assumed to reflect genomic changes at large (CNV) and small (SNP) scales. Comparisons of data type effects at all marker sizes are best seen in Figure 4.

The transition from normal tissue to cancerous tissue is generally associated with changes at the level of both gene expression and the genome. Frequent mutations, genomic rearrangements and large scale changes in gene expression are all characteristic of oncogenic transformation. However, cancer cells also retain many, if not most, of the essential hallmarks of the tissue of origin of the cancer. In this study, we use the tissue of origin as the *ground truth *and measure the ability of each data type to correctly infer the tissue of origin of a sample based upon each data type.

*A priori*, we expect some of these data types to be better at this task than others. For instance, mRNA profiles are highly distinct between different tissue types. For this reason, even after oncogenic transformation, an mRNA transcriptional profile characteristic of the tissue of origin is expected to resemble that of the normal tissue, more than it would the transcriptional profile of tumors derived from other tissues. For this reason, we expect (and find) that mRNA transcriptional profiles reliably and accurately infer the tissue of origin of tumor cell lines. Similarly, protein expression profiles are also very reliable indicators of the tissue of origin of a tumor. microRNA profiles are less powerful than either mRNA or protein expression profiles, but still fairly powerful indicators of tissue of origin. The relative weakness of microRNA profiles compared to mRNA and protein expression profiles may in part result from lower tissue specificity of microRNA expression relative to mRNA and protein.

The ability of genomic data to infer the tissue of origin of the tumor is subject to a very different set of biological constraints than expression data. While expression data is expected to be approximately identical across tissues regardless of patient identity, and thus similar between tumors derived from the same tissue but from different individuals; genomic data is identical across normal tissues in an individual, and differs between individuals. Thus, at first glance, genomic data would be expected to track with the individual, and be a very poor predictor of the tissue of origin of a cancer. However, dramatic genomic alterations are a hallmark of cancer progression, and distinct genomic alterations are often found in distinct cancer types. Accordingly, we find that copy number variation is about as powerful as microRNA profiles at inferring the tissue of origin of a cancer cell. This is likely due to the preferential occurrence of specific DNA rearrangements in cancers derived from specific cell types [37]. The SNP arrays however, which measure the presence of specific alleles in a sample, show unexpectedly strong ability to infer the tissue of origin of these cancer cell lines. Indeed, their performance is similar to that of the mRNA and protein expression profiles (perhaps even better at high numbers of markers). This was unexpected as SNP's should be roughly identical across all tissues in an individual, and by and large, reflect an individual's ancestry. However, this phenomenon has been previously observed in the NCI-60 data, and was found to result from the fact that intensity of signal on the SNP array was actually reflecting SNP copy number at duplicated loci, and thus indirectly measuring likely gene expression levels, rather than homogenization of genotypic diversity [38]. This effect was strongest for linked SNPs, and appears to be the result of local gene copy number amplification, which in turn enables increased gene expression. Thus, the ability of SNP arrays to accurately infer tissue of origin of cancer cell lines appears to result from increased gene expression driven by local duplication and increase in copy number. As the CGH arrays used to profile the NCI-60 lines provide much lower genomic resolution than the SNP arrays, they are less powerful at detecting and exploiting this effect. This unexpected behavior of the SNP arrays used to characterize the NCI-60 lines could be addressed by utilizing newer SNP arrays that control for copy number such as the Affymetrix SNP6 platform.

### Number of marker effects

As one uses more biomarkers to classify samples, one expects increased performance, the possibility of overfitting, and the appearance of a plateau beyond which additional markers do not increase the power of classification. However, the rate at which these changes occur as more markers are used to classify a sample can be very different for various types of data.

Our analysis shows that mRNA, protein, and SNP data all plateau at about the same AUC (*∼*0.97).

However, each of these data types reaches the plateau at a different number of markers: mRNA plateaus between 16 and 32 markers, while protein plateaus at around 32 markers, and SNP does not reach the same AUC until 64 markers are used. This may result from the fact that each of these markers appear to measuring aspects of gene expression, with decreasing directness (SNP) or coverage (protein), and thus power of discrimination. The mRNA arrays used to characterize the NCI-60 cell lines provide direct assessment of the activity of thousands of protein-coding genes, while the protein arrays measure only somewhat more than 300 proteins. With thousands of potential markers to choose from, the mRNA-based models can select informative markers from a larger marker pool, and thus maximize the performance of a gene expression-based model more quickly than the protein arrays, which are restricted to a small subset of the protein coding genes represented on the mRNA arrays. The more direct nature of the protein measurement (i.e. closer to the active biological component) does not appear to outweigh the disadvantage of the lower coverage in the starting set of protein markers. As discussed in the preceding section, the SNP array appears to be measuring, in part, gene expression levels resulting from the amplification of specific regions of the genome in specific cancer types. However, there is likely to be a complex and possibly heterogeneous and non-linear relationship between signal intensity on the SNP array, and gene expression levels. Thus, despite the very large number of markers to choose from on the SNP array, highly informative markers are not as abundant in this data as they appear to be in the mRNA data. As a result, many more SNP markers are required to achieve the same level of performance as mRNA-based markers. It is hard to predict how the power of SNPs to infer cancer type might change when newer arrays, that control for copy number changes, are used to characterize these cell lines.

Similarly, CNV and microRNA markers approach the same level of performance as one another, but do so at different rates. While microRNA markers plateau quickly (at about 16 markers) CNV markers require 64-96 markers to reach the same level of performance. The quick plateau of microRNA-based markers is likely due to the highly tissue-specific expression of a minority of microRNA's, and the more global expression of the remaining majority. Once the few highly informative microRNA's have been selected and used, adding more provides little additional classification power. In the case of CNV's, like SNP's these markers reflect changes in the cancer cells genome that can lead to changes in gene expression that are distinctive features of cancer subtypes. However, not only do the CNV markers suffer from the indirect relationship between the marker and gene expression expected for SNP's, they are also a much lower resolution marker than SNP's (megabases vs single bases), and far fewer CNV's were measured on the arrays, thus limiting the likelihood that the most informative CNV's were available for selection. Thus, the power of the CNV biomarker panel climbs slowly.

Taken together, these observations suggest that the absolute performance of a given biomarker data type to classify a cancer can be understood in the context of: the number of available markers for the model to choose from, the power of the most informative markers in the set, and the directness with which the data type reflects an informative aspect of the sample biology. Data types with a large number of markers to choose from, that are closely related to the biology of the sample, are most likely to yield highly effective small biomarker panels. On another hand, data types with lower saturation (fewer markers measured), and/or a less direct relationship to the biological differences between samples, will require more markers to reach maximal performance.

### Combined model, data type, and number of marker effects

Ultimately all parameters should be considered simultaneously when attempting to build the best targeted predictive model. In order to do this it is necessary to build a validation framework to explore all parameters fairly and efficiently. Although it is a difficult task it is not impractical and interesting nuances can be extracted.

In this study it was observed that at the lowest number of markers (8) mRNA and protein were the best data types for cancer identification. For mRNA, SNP and protein the SVM-RFE was the best feature selection choice and ET was the best classifier. For CNV and microRNA the best classifier was LR and ET respectively. Interestingly for all data types at 8 markers except CNV a tree based classifier performed the best as seen in Table 1. It is possible that if only a few biomarkers are considered the tree based approaches explicit enumeration of decisions may be better suited, however it should be noted that the linear classifiers are typically only marginally worse.

At the highest number of markers tests (96) both RFE and ET perform strongly on all data types, however LR is the best classifier for all types except SNP where KNN is the best. Both of these classification tools are technically simple, yet they perform best which lends credence to the Occam's razor principle which when applied to machine learning places preference on simpler explanations.

### External validation

The amount of over-fitting when building a predictive model is always a concern. This effect was measured in an external validation experiment utilizing analogous gene expression microarray data obtained from several studies which are part of the TCGA project [26-34]. The results of this comparison indicated that biomarker and model selection using AUC as the ranking criteria is robust and performs well across studies. In Table 2 it can be seen that colon (CO), CNS and renal (RE) cancer types were distinguishable with a high degree of accuracy using between 8 and 96 markers. The CNS type was more challenging to differentiate after 32 markers, while ovarian (OV) and lung cancers (LC) were extremely difficult to differentiate at any number of markers.

**Table 2:** External validation accuracy by cancer type.

Marker Set Size	CO	OV	CNS	LC	RE
8	0.1673	0	1	0.3656	0.0138
16	0.9856	0.037	0.8246	0.686	0.7403
32	1	0.1111	0.9123	0.2384	0.8571
64	1	0.0741	0.5965	0.1163	0.8961
96	1	0.2593	0.5351	0.0116	1

Accuracy of the top performing model for each cancer type and grouped by marker set size.

The NCI-60 data is derived from decades old cell lines, while the TCGA data was derived from recently sampled primary solid tumors. Additionally the matched cancer types did not have comparable histological classification. Finally, there were three aditional cancer types (ME, LE, BR) which were present in NCI-60 but not included in the external validation set. These classes were included in the training. Despite these differences the presented method was able to perform biomarker selection and build accurate predictive models for this challenging external validation experiment. A complete breakdown of the per-class prediction rate by cancer marker set size is provided in Supplemental Table 9.

## Conclusions

The initial hypothesis motivating this research was that certain predictive models will perform better on different data types at different dimensionalities. While this hypothesis holds, the difference in accuracy between models is often small and allows for several generalizations. Namely that RFE is clearly the best feature selection algorithm and both SVM and LR are the best classifiers as seen in Figures 2 and 3. Both mRNA and protein expression are the overall best performing data types for the cancer classification question. However to maximize predictive accuracy all models at all parameters should be parameterized and vetted fairly before conclusions are made.

## List of abbreviations

NCI-60. National Cancer Institutes tumor cell line project; TCGA. The Cancer Genome Atlas; SVM. Support vector machine; DT. Decision tree; RF. Random forest; ET. Extra tree; GB. Gradient boosting; KNN. K-nearest neighbor; RFE. Recursive feature elimination; LR. Logistic regression; COS. Cosine; COR. Correlation; BR. Breast; CNS. Central nervous system; CO. Colon; LC. Lung cancer; RE. Renal; OV. Ovarian; ME. Melanoma; SNP. Single nucleotide polymorphism

## Competing interests

IIM, CEN, EH and JL are co-founders and own shares of Smpl Bio LLC a for-profit business created to commercialize the application of the work presented here. Smpl Bio LLC has not contributed financially to this publication. The University of Connecticut has filed a patent application on work related to this publication.

## Authors' contributions

IIM and CEN conceived the study. EH, JL, and CL conducted the experiments. All the authors contributed valuable ideas to the study, drafted the manuscript, and were involved in manuscript revision. All authors have read and approved the final manuscript.

## Supplementary Material

Additional file 1 **Supplementary figures, methods, and tables are supplied in PDF format**. Click here for file

## Declarations

Publication costs for this work were funded by the corresponding authors' institution.

This article has been published as part of *BMC Bioinformatics *Volume 15 Supplement 13, 2014: Selected articles from the 9th International Symposium on Bioinformatics Research and Applications (ISBRA'13): Bioinformatics. The full contents of the supplement are available online at http://www.biomedcentral.com/bmcbioinformatics/supplements/15/S13.
