# Supplementary material for: Feature selection and classifier performance on diverse bio- logical datasets
Source: BMC Bioinformatics. 2014 Nov 13;15(Suppl 13):S4. doi: 10.1186/1471-2105-15-S13-S4 (PMC4248652; doi:10.1186/1471-2105-15-S13-S4)
Supplement: Additional file 1 — Supplementary figures, methods, and tables are supplied in PDF format. [file 1471-2105-15-S13-S4-S1.pdf]

Additional file 1 — Supplementary methods

The eight NCI-60 cell-lines were used as the training data for marker set sizes 8, 16, 32, 64, and 96. The nine primary tumor cohorts from the TCGA data set were normalized to the NCI-60 data set using quantile normalization using NCI-60 data as the target distribution.

As discussed in the Validation Strategy section, a nested 5-fold cross validation with repeating the outer cross validation 100 times was utilized to train the NCI-60 data set, using the same feature selection and classifier methods described in the main text. Before running the nested k-fold cross validation, the training data was filtered to the top 1,000 features ranked using the F-statistic. The resulting top models used for feature selection and classification is shown in Supplementary Table **??**. For each marker set size, the NCI-60 training data is used to extract the features and used as the reference data set for classification. The resulting accuracy of the validation experiment is defined as the number of correct predictions divided by the number of total predictions.

Additional file 1 — Supplementary tables

Table 1: Datatypes Downloaded from CellMiner

Data Type	CellMiner Data Type	Normalization Option	CellMiner File Name
SNP	DNA: Affy 500K SNP	CRLMM	nci60_DNA__Affy_500K_SNP_CRLMM.txt.zip
mRNA	RNA: Affy HuEx 1.0	GCRMA	nci60_RNA__Affy_HuEx_1.0_GCRMA.txt.zip
CNV	DNA: aCGH Agilent 44K	AgelentFE	nci60_DNA__aCGH_Agilent_44K_AgilentFE.txt.zip
microRNA	RNA: microRNA OSU V3 chip	log2	nci60_RNA__microRNA_OSU_V3_chip_log2.txt.zip
Protein	Protein: Lysate Array	log2	nci60_Protein__Lysate_Array_log2.txt.zip

Data files and normalization strategy used for each data set

Table 2: Cell Lines used in Analysis

**Data Type**	**Breast**	**CNS**	**Colon**	**Non-Small Cell Lung**	**Leukemia**	**Melanoma**	**Ovarian**	**Renal**
mRNA	BR.MCF7<br>BR.MDA_MB_231<br>BR.HS578T<br>BR.BT_549<br>BR.T47D	CNS.SF_268<br>CNS.SF_295<br>CNS.SNB_19<br>CNS.SNB_75<br>CNS.U251	CO.COLO205<br>CO.HCC_2998<br>CO.HCT_116<br>CO.HCT_15<br>CO.HT29<br>CO.KM12<br>CO.SW_620	LC.A549<br>LC.EKVX<br>LC.HOP_62<br>LC.HOP_92<br>LC.NCI_H226<br>LC.NCI_H23<br>LC.NCI_H322M<br>LC.NCI_H460<br>LC.NCI_H522	LE.CCRF_CEM<br>LE.HL_60<br>LE.K_562<br>LE.MOLT_4<br>LE.RPMI_8226<br>LE.SR	ME.LOXIMVI<br>ME.MALME_3M<br>ME.M14<br>ME.SK_MEL_2<br>ME.SK_MEL_28<br>ME.SK_MEL_5<br>ME.UACC_257<br>ME.UACC_62<br>ME.MDA_MB_435<br>ME.MDA_N	OV.IGROV1<br>OV.OVCAR_3<br>OV.OVCAR_4<br>OV.OVCAR_5<br>OV.OVCAR_8<br>OV.SK_OV_3<br>OV.NCI_ADR_RES	RE.786_0<br>RE.A498<br>RE.ACHN<br>RE.CAKI_1<br>RE.RXF_393<br>RE.SN12C<br>RE.TK_10<br>RE.UO_31
Protein	BR.MCF7<br>BR.MDA_MB_231<br>BR.HS578T<br>BR.BT_549<br>BR.T47D	CNS.SF_268<br>CNS.SF_295<br>CNS.SF_539<br>CNS.SNB_19<br>CNS.SNB_75<br>CNS.U251	CO.COLO205<br>CO.HCC_2998<br>CO.HCT_116<br>CO.HCT_15<br>CO.HT29<br>CO.KM12<br>CO.SW_620	LC.A549<br>LC.EKVX<br>LC.HOP_62<br>LC.HOP_92<br>LC.NCI_H226<br>LC.NCI_H23<br>LC.NCI_H322M<br>LC.NCI_H460<br>LC.NCI_H522	LE.CCRF_CEM<br>LE.HL_60<br>LE.K_562<br>LE.MOLT_4<br>LE.RPMI_8226<br>LE.SR	ME.LOXIMVI<br>ME.MALME_3M<br>ME.M14<br>ME.SK_MEL_2<br>ME.SK_MEL_28<br>ME.SK_MEL_5<br>ME.UACC_257<br>ME.UACC_62<br>ME.MDA_MB_435<br>ME.MDA_N	OV.IGROV1<br>OV.OVCAR_3<br>OV.OVCAR_4<br>OV.OVCAR_5<br>OV.OVCAR_8<br>OV.SK_OV_3<br>OV.NCI_ADR_RES	RE.786_0<br>RE.A498<br>RE.ACHN<br>RE.CAKI_1<br>RE.RXF_393<br>RE.SN12C<br>RE.TK_10<br>RE.UO_31
microRNA	BR.MCF7<br>BR.MDA_MB_231<br>BR.HS578T<br>BR.BT_549<br>BR.T47D	CNS.SF_268<br>CNS.SF_295<br>CNS.SF_539<br>CNS.SNB_19<br>CNS.SNB_75<br>CNS.U251	CO.COLO205<br>CO.HCC_2998<br>CO.HCT_116<br>CO.HCT_15<br>CO.HT29<br>CO.KM12<br>CO.SW_620	LC.A549<br>LC.EKVX<br>LC.HOP_62<br>LC.HOP_92<br>LC.NCI_H226<br>LC.NCI_H23<br>LC.NCI_H322M<br>LC.NCI_H460<br>LC.NCI_H522	LE.CCRF_CEM<br>LE.HL_60<br>LE.K_562<br>LE.MOLT_4<br>LE.RPMI_8226<br>LE.SR	ME.LOXIMVI<br>ME.MALME_3M<br>ME.M14<br>ME.SK_MEL_2<br>ME.SK_MEL_28<br>ME.SK_MEL_5<br>ME.UACC_257<br>ME.UACC_62<br>ME.MDA_MB_435<br>ME.MDA_N	OV.IGROV1<br>OV.OVCAR_3<br>OV.OVCAR_4<br>OV.OVCAR_5<br>OV.OVCAR_8<br>OV.SK_OV_3<br>OV.NCI_ADR_RES	RE.786_0<br>RE.A498<br>RE.ACHN<br>RE.CAKI_1<br>RE.RXF_393<br>RE.SN12C<br>RE.TK_10<br>RE.UO_31
SNP	BR.MCF7<br>BR.MDA_MB_231<br>BR.HS578T<br>BR.BT_549<br>BR.T47D	CNS.SF_268<br>CNS.SF_295<br>CNS.SF_539<br>CNS.SNB_19<br>CNS.SNB_75<br>CNS.U251	CO.COLO205<br>CO.HCC_2998<br>CO.HCT_116<br>CO.HCT_15<br>CO.HT29<br>CO.KM12<br>CO.SW_620	LC.A549<br>LC.EKVX<br>LC.HOP_62<br>LC.HOP_92<br>LC.NCI_H226<br>LC.NCI_H23<br>LC.NCI_H322M<br>LC.NCI_H460<br>LC.NCI_H522	LE.CCRF_CEM<br>LE.HL_60<br>LE.K_562<br>LE.MOLT_4<br>LE.RPMI_8226<br>LE.SR	ME.LOXIMVI<br>ME.MALME_3M<br>ME.M14<br>ME.SK_MEL_2<br>ME.SK_MEL_28<br>ME.SK_MEL_5<br>ME.UACC_257<br>ME.UACC_62<br>ME.MDA_MB_435<br>ME.MDA_N	OV.IGROV1<br>OV.OVCAR_3<br>OV.OVCAR_4<br>OV.OVCAR_5<br>OV.OVCAR_8<br>OV.SK_OV_3<br>OV.NCI_ADR_RES	RE.786_0<br>RE.A498<br>RE.ACHN<br>RE.CAKI_1<br>RE.RXF_393<br>RE.SN12C<br>RE.TK_10<br>RE.UO_31
CNV	BR.MCF7<br>BR.MDA_MB_231<br>BR.HS578T<br>BR.BT_549<br>BR.T47D	CNS.SF_268<br>CNS.SF_295<br>CNS.SF_539<br>CNS.SNB_19<br>CNS.SNB_75<br>CNS.U251	CO.COLO205<br>CO.HCC_2998<br>CO.HCT_116<br>CO.HCT_15<br>CO.HT29<br>CO.KM12<br>CO.SW_620	LC.A549<br>LC.EKVX<br>LC.HOP_62<br>LC.HOP_92<br>LC.NCI_H226<br>LC.NCI_H23<br>LC.NCI_H322M<br>LC.NCI_H460<br>LC.NCI_H522	LE.CCRF_CEM<br>LE.HL_60<br>LE.K_562<br>LE.MOLT_4<br>LE.RPMI_8226<br>LE.SR	ME.LOXIMVI<br>ME.MALME_3M<br>ME.M14<br>ME.SK_MEL_2<br>ME.SK_MEL_28<br>ME.SK_MEL_5<br>ME.UACC_257<br>ME.UACC_62<br>ME.MDA_MB_435<br>ME.MDA_N	OV.IGROV1<br>OV.OVCAR_3<br>OV.OVCAR_4<br>OV.OVCAR_5<br>OV.OVCAR_8<br>OV.SK_OV_3<br>OV.NCI_ADR_RES	RE.786_0<br>RE.A498<br>RE.ACHN<br>RE.SN12C<br>RE.TK_10<br>RE.UO_31

The cell lines used in the cross validation experiment.

Table 3: Feature Selection Parameters

FS Method	Parameter	Description	Values
Anova	NA	No parameters	
RFE	Estimator	The supervised learning estimator	Support Vector Classification (SVC) with a linear kernal for the decision functions
	Estimator Parameter - C	The penalty parameter of the error term	0.25, 1, 4, 16, 64, 256
Random Forest	Max Features	Function to determine the number of features to consider when looking for best split	sqrt and log2
	N Estimators	The number of trees to be used in the forest	10, 50, 100, 250
Extra Trees	Max Features	Function to determine the number of features to consider when looking for best split	sqrt and log2
	N Estimators	The number of trees to be used in the forest	10, 50, 100, 250

The tested parameters for each feature selection algorithm.

Table 4: Classification Parameters

CL Method	Parameter	Description	Values
Support Vector Machine	Kernel	Function to use as a decision function	RBF and Linear
	RBF Gamma	Kernel coefficient for RBF	0.03125, 0.125, 0.5, 2, 8, 32, 128, and 512
	RBF C	Penalty Parameter of the error term	0.03125, 0.125, 0.5, 2, 8, 32, 128, and 512
	Linear C	Penalty Parameter of the error term	0.03125, 0.125, 0.5, 2, 8, 32, 128, and 512
Logistic Regression	Penalty	Norm used in the penalization	l2
	C	Inverse of regularization strength; smaller values specify stronger regularization	0.25, 1, 4, 16, 64, and 256
Decision Trees	Max Features	Function to determine the number of features to consider when looking for best split	sqrt and log2
Random Forest	Max Features	Function to determine the number of features to consider when looking for best split	sqrt and log2
	N Estimators	Number of trees to be used in the forest	10, 50, 100, 250
Extra Trees	Max Features	Function to determine the number of features to consider when looking for best split	sqrt and log2
	N Estimators	Number of trees to be used in the forest	10, 50, 100, 250
Gradient Boosting	N Boosting Stages	Number of boosting stages to perform	100, 250, and 500
	Max Depth	Maximum depth of the individual regression estimators	3, 5, 7
K-nearest Neighbors	Compute Nearest Neighbor	Algorithm used to compute the nearest neighbors	BallTree and KDtree
	Distance Function	Function used to calculate the distance	Euclidean and Manhattan
	Weight Function	A function to apply weights to the points	Uniform and Inverse weighting based on the distance to their neighbors; the closer the distance the better the score.
Cosine	NA	No parameters	
Correlation	NA	No parameters	

The tested parameters for each classification algorithm.

Table 5: Mean AUC per Model Grouped by Platform and Marker Set Size

Marker Set Size	mRNA			Protein			microRNA			SNP			CNV		
**8**	**RFE**	**ET**	**0.9585**	**RFE**	**ET**	**0.9426**	**RFE**	**RF**	**0.8352**	**RFE**	**ET**	**0.8598**	**RFE**	**LR**	**0.7198**
	RFE	RF	0.9554	ET	ET	0.9394	RFE	SVM	0.8352	RFE	RF	0.8591	ET	LR	0.7115
	RFE	SVM	0.9521	RFE	RF	0.9382	RFE	KNN	0.8295	RFE	SVM	0.8321	RF	LR	0.71
	RFE	LR	0.951	RF	ET	0.9376	RFE	ET	0.8275	ET	ET	0.8295	RFE	ET	0.691
	RFE	KNN	0.9467	ET	RF	0.9312	Anova	SVM	0.8089	ET	RF	0.8247	RFE	RF	0.6802
	RFE	Cos	0.9397	RF	RF	0.9272	Anova	LR	0.8051	RF	ET	0.8177	RFE	SVM	0.6702
	RFE	Corr	0.9299	RFE	SVM	0.922	RF	ET	0.8028	RF	RF	0.817	ET	ET	0.6698
	ET	LR	0.9114	RF	SVM	0.9187	RF	RF	0.8027	RFE	KNN	0.8161	RFE	KNN	0.6681
	RF	LR	0.9113	ET	SVM	0.9177	RFE	LR	0.8021	RF	LR	0.816	ET	RF	0.6648
	RF	ET	0.9093	RF	LR	0.9168	RF	LR	0.802	RFE	LR	0.8138	RF	ET	0.6631
	RFE	GB	0.9076	ET	LR	0.9165	RFE	GB	0.7962	ET	LR	0.8114	RF	RF	0.663
	ET	ET	0.9061	ET	KNN	0.9161	RF	SVM	0.7951	ET	SVM	0.8006	ET	KNN	0.6543
	ET	RF	0.9029	RF	KNN	0.9156	ET	RF	0.7947	RF	SVM	0.7955	RFE	GB	0.6528
	RF	RF	0.9006	Anova	ET	0.9076	ET	ET	0.7883	ET	KNN	0.7826	RF	KNN	0.6463
	RF	SVM	0.8969	RFE	KNN	0.9039	RF	KNN	0.7878	RFE	Cos	0.7719	ET	SVM	0.6304
	ET	SVM	0.8875	RFE	LR	0.8986	RFE	Cos	0.7867	RF	KNN	0.7712	RF	GB	0.6292
	ET	KNN	0.8806	Anova	RF	0.8925	Anova	ET	0.7867	RFE	Corr	0.7698	RF	SVM	0.6217
	RF	KNN	0.8788	Anova	LR	0.8808	Anova	RF	0.7848	Anova	RF	0.7659	ET	GB	0.6188
	ET	GB	0.8738	Anova	KNN	0.8701	RFE	Corr	0.7812	Anova	ET	0.7604	Anova	LR	0.5883
	RF	Cos	0.8647	RFE	Cos	0.8693	Anova	KNN	0.7806	Anova	SVM	0.753	RFE	Corr	0.5756
	RF	GB	0.8619	Anova	SVM	0.8598	RF	Corr	0.7762	RF	GB	0.7289	Anova	KNN	0.5722
	ET	Cos	0.8603	RF	Cos	0.852	ET	KNN	0.7738	ET	Corr	0.7239	RF	Corr	0.5721
	RF	Corr	0.8518	ET	Cos	0.8502	ET	SVM	0.7724	ET	Cos	0.722	RFE	DT	0.5663
	ET	Corr	0.8475	RFE	Corr	0.8381	Anova	Corr	0.7722	ET	GB	0.7197	ET	Corr	0.5646
	RFE	DT	0.7807	ET	Corr	0.8202	Anova	Cos	0.7662	Anova	LR	0.7179	Anova	ET	0.5637
	ET	DT	0.7325	RF	Corr	0.8132	RF	Cos	0.7624	RF	Cos	0.7172	Anova	RF	0.5636
	RF	DT	0.7319	ET	GB	0.8132	ET	LR	0.7602	Anova	KNN	0.7166	ET	DT	0.5628
	Anova	SVM	0.6732	RF	GB	0.8101	ET	GB	0.7537	RFE	GB	0.7146	RF	DT	0.5587
	Anova	ET	0.6588	RFE	GB	0.8062	RF	GB	0.7487	RF	Corr	0.701	Anova	SVM	0.5549
	Anova	RF	0.651	Anova	Cos	0.7996	Anova	GB	0.7484	Anova	Corr	0.6686	Anova	GB	0.554
	Anova	LR	0.6368	Anova	GB	0.7656	ET	Corr	0.7441	Anova	Cos	0.6673	RFE	Cos	0.5457
	Anova	Corr	0.6191	Anova	Corr	0.7562	ET	Cos	0.7422	RFE	DT	0.6321	RF	Cos	0.5424
	Anova	KNN	0.6184	RFE	DT	0.7197	RFE	DT	0.6584	Anova	GB	0.6317	ET	Cos	0.5392
	Anova	GB	0.6146	RF	DT	0.7152	Anova	DT	0.6473	RF	DT	0.6195	Anova	DT	0.5343
	Anova	Cos	0.6134	ET	DT	0.7115	ET	DT	0.6389	ET	DT	0.6173	Anova	Corr	0.5213
	Anova	DT	0.5647	Anova	DT	0.6799	RF	DT	0.6385	Anova	DT	0.5769	Anova	Cos	0.5164
**16**	**RFE**	**ET**	**0.972**	**RFE**	**ET**	**0.9666**	**RFE**	**SVM**	**0.8758**	**RFE**	**ET**	**0.922**	**ET**	**LR**	**0.7616**
	RFE	LR	0.9709	ET	ET	0.9582	RFE	KNN	0.8704	RFE	RF	0.9162	RFE	LR	0.7607
	RFE	RF	0.9681	RFE	RF	0.9565	RFE	RF	0.8671	RFE	SVM	0.9111	RF	LR	0.7468
	RFE	SVM	0.968	RF	ET	0.9539	RFE	ET	0.8597	RFE	KNN	0.9033	RFE	ET	0.7159
	RFE	Cos	0.9663	ET	RF	0.9482	RFE	LR	0.8535	ET	ET	0.8997	RFE	RF	0.7086
	RFE	KNN	0.9604	ET	KNN	0.9435	Anova	SVM	0.8496	RFE	LR	0.897	RFE	SVM	0.7067
	RF	LR	0.9588	RF	RF	0.9414	Anova	LR	0.8439	RF	ET	0.896	ET	ET	0.7009
	RFE	Corr	0.9576	RF	KNN	0.9413	RFE	Cos	0.8407	ET	RF	0.8914	ET	RF	0.7007
	RF	ET	0.9514	ET	SVM	0.9407	Anova	RF	0.8361	RF	RF	0.8861	RF	ET	0.6995
	ET	LR	0.9508	RF	SVM	0.9381	Anova	KNN	0.8349	RF	LR	0.878	RF	RF	0.699
	ET	ET	0.9482	RFE	SVM	0.938	RFE	Corr	0.8325	ET	LR	0.8767	RFE	GB	0.6899
	ET	RF	0.9468	ET	LR	0.9305	Anova	ET	0.8323	ET	SVM	0.8766	ET	SVM	0.6896
	RF	RF	0.9453	Anova	ET	0.9302	RF	LR	0.8295	RF	SVM	0.8675	ET	KNN	0.6827
	RF	SVM	0.9393	RFE	KNN	0.925	ET	RF	0.8273	ET	KNN	0.8593	RF	SVM	0.6799
	RFE	GB	0.9306	Anova	RF	0.9193	RF	ET	0.8265	RFE	Corr	0.8591	RF	KNN	0.6743
	RF	KNN	0.9242	RF	LR	0.9192	RF	KNN	0.8256	RF	KNN	0.8545	RFE	KNN	0.6739
	ET	KNN	0.9242	RFE	LR	0.9086	Anova	Corr	0.8252	RFE	Cos	0.8508	ET	GB	0.6585
	ET	SVM	0.9233	Anova	LR	0.9076	RF	RF	0.8243	Anova	RF	0.8142	RF	GB	0.6566
	RF	Cos	0.923	Anova	KNN	0.902	RF	SVM	0.8234	Anova	ET	0.7982	RFE	Corr	0.621
	RF	Corr	0.9224	Anova	SVM	0.8914	Anova	Cos	0.8226	ET	Corr	0.7898	Anova	LR	0.6089
	ET	GB	0.915	RFE	Cos	0.8871	RF	Corr	0.8212	ET	Cos	0.7885	RFE	Cos	0.6075
	RF	GB	0.9145	ET	Cos	0.8836	ET	ET	0.8156	RF	Cos	0.7812	RF	Corr	0.6066
	ET	Cos	0.9087	RF	Cos	0.8744	ET	KNN	0.8137	Anova	SVM	0.7779	ET	Corr	0.6061
	ET	Corr	0.906	RFE	Corr	0.8641	RFE	GB	0.8058	RF	Corr	0.7717	Anova	KNN	0.5878
	RFE	DT	0.8129	ET	Corr	0.8618	ET	SVM	0.803	RF	GB	0.7596	Anova	RF	0.5829
	ET	DT	0.794	RF	Corr	0.8552	ET	LR	0.7982	Anova	LR	0.7511	RFE	DT	0.5768
	RF	DT	0.7857	Anova	Cos	0.8424	RF	Cos	0.7968	ET	GB	0.7426	Anova	ET	0.5764
	Anova	SVM	0.7232	Anova	Corr	0.8266	ET	Corr	0.7945	Anova	KNN	0.733	Anova	SVM	0.575
	Anova	ET	0.7192	ET	GB	0.8189	ET	Cos	0.7897	RFE	GB	0.7078	RF	DT	0.5726
	Anova	LR	0.7157	RFE	GB	0.8169	Anova	GB	0.7858	Anova	Cos	0.6791	Anova	GB	0.57
	Anova	RF	0.705	RF	GB	0.8069	ET	GB	0.7715	Anova	Corr	0.6732	ET	DT	0.5688
	Anova	Corr	0.6893	Anova	GB	0.7795	RF	GB	0.7609	Anova	GB	0.6581	RF	Cos	0.5569
	Anova	KNN	0.6761	ET	DT	0.7277	RFE	DT	0.674	RF	DT	0.6481	ET	Cos	0.5552
	Anova	Cos	0.6749	RFE	DT	0.725	Anova	DT	0.6684	RFE	DT	0.6396	Anova	DT	0.5402
	Anova	GB	0.6646	RF	DT	0.716	ET	DT	0.6563	ET	DT	0.638	Anova	Corr	0.5335
	Anova	DT	0.5911	Anova	DT	0.6945	RF	DT	0.6529	Anova	DT	0.6007	Anova	Cos	0.5163
**32**	**RFE**	**LR**	**0.9759**	**RFE**	**ET**	**0.9792**	**RFE**	**KNN**	**0.8806**	**RFE**	**LR**	**0.9685**	**RFE**	**LR**	**0.8194**
	RFE	ET	0.9757	ET	ET	0.969	RFE	RF	0.8801	RFE	SVM	0.9674	ET	LR	0.7904
	RF	LR	0.9747	RFE	RF	0.969	RFE	ET	0.8717	RFE	KNN	0.966	RF	LR	0.788
	RFE	Cos	0.9736	RFE	KNN	0.9689	RFE	SVM	0.8679	RFE	ET	0.9646	RFE	SVM	0.7607
	RFE	RF	0.9734	RF	ET	0.9645	RFE	LR	0.866	RFE	RF	0.9577	RFE	ET	0.743
	RFE	SVM	0.9734	Anova	ET	0.9636	Anova	LR	0.8538	ET	ET	0.9518	ET	ET	0.7278
	ET	LR	0.9683	ET	KNN	0.9617	Anova	SVM	0.8528	RF	ET	0.9504	RFE	RF	0.7261
	RF	ET	0.9676	RFE	SVM	0.9602	RFE	Corr	0.8525	RF	LR	0.9477	RF	ET	0.7259
	RFE	KNN	0.9672	ET	RF	0.9594	Anova	KNN	0.851	RF	RF	0.9448	RFE	KNN	0.7257
	RF	RF	0.9666	ET	SVM	0.9587	RFE	Cos	0.85	ET	LR	0.9438	RF	RF	0.7209
	RFE	Corr	0.9662	RF	KNN	0.9583	RF	KNN	0.8479	ET	RF	0.9412	ET	SVM	0.7206
	ET	RF	0.9658	Anova	KNN	0.9573	RF	Corr	0.8457	RF	SVM	0.9395	RF	SVM	0.7196
	ET	ET	0.9625	RF	SVM	0.9562	RF	LR	0.8435	ET	SVM	0.9384	ET	RF	0.7159
	RF	SVM	0.9543	Anova	RF	0.9559	RF	RF	0.8408	RF	KNN	0.9352	RFE	GB	0.7097
	RF	Cos	0.9541	RF	RF	0.9547	Anova	RF	0.8406	ET	KNN	0.9312	RF	KNN	0.7088
	RF	Corr	0.9524	ET	LR	0.9543	ET	RF	0.8402	RFE	Corr	0.9225	ET	KNN	0.7034
	RF	KNN	0.9462	RFE	LR	0.9537	RF	ET	0.8395	RFE	Cos	0.9122	RFE	Cos	0.6879
	ET	SVM	0.9439	Anova	LR	0.9532	ET	KNN	0.8381	Anova	RF	0.8604	ET	GB	0.6712
	ET	KNN	0.9438	Anova	SVM	0.9505	Anova	Corr	0.8362	ET	Corr	0.8601	RFE	Corr	0.669
	ET	Cos	0.9424	RF	LR	0.9479	RF	SVM	0.8303	ET	Cos	0.8589	RF	GB	0.6665
	RFE	GB	0.9414	ET	Cos	0.9015	Anova	ET	0.83	RF	Cos	0.8565	RF	Corr	0.6321
	ET	Corr	0.9405	Anova	Cos	0.9007	Anova	Cos	0.8291	RF	Corr	0.8503	Anova	LR	0.6272
	ET	GB	0.9397	RFE	Cos	0.8979	ET	LR	0.8282	Anova	ET	0.8425	ET	Corr	0.6218

Continued on next page

– Continued from previous page

Marker Set Size	mRNA			Protein			microRNA			SNP			CNV		
	RF	GB	0.9386	Anova	Corr	0.8922	ET	Corr	0.8266	Anova	SVM	0.8053	RF	Cos	0.592
	RFE	DT	0.8215	RF	Cos	0.8918	ET	ET	0.8257	Anova	LR	0.7989	Anova	RF	0.5902
	Anova	LR	0.8094	ET	Corr	0.8891	RF	Cos	0.824	RF	GB	0.7812	Anova	KNN	0.5898
	RF	DT	0.8039	RF	Corr	0.8803	ET	SVM	0.8229	ET	GB	0.765	Anova	SVM	0.5838
	ET	DT	0.8033	RFE	Corr	0.8797	ET	Cos	0.8186	Anova	KNN	0.7539	Anova	ET	0.5825
	Anova	SVM	0.7995	ET	GB	0.8238	Anova	GB	0.7926	Anova	GB	0.7014	RFE	DT	0.5797
	Anova	ET	0.7935	RF	GB	0.8109	RFE	GB	0.7897	RFE	GB	0.6999	Anova	GB	0.5796
	Anova	RF	0.7844	Anova	GB	0.8088	ET	GB	0.7728	Anova	Cos	0.6932	ET	Cos	0.5767
	Anova	Cos	0.7654	RFE	GB	0.8087	RF	GB	0.7563	Anova	Corr	0.6861	RF	DT	0.5741
	Anova	Corr	0.7636	ET	DT	0.723	RFE	DT	0.6703	RF	DT	0.6541	ET	DT	0.5713
	Anova	KNN	0.745	RFE	DT	0.7204	Anova	DT	0.6658	ET	DT	0.6461	Anova	Corr	0.5474
	Anova	GB	0.7424	RF	DT	0.7189	ET	DT	0.6546	RFE	DT	0.6445	Anova	DT	0.5452
	Anova	DT	0.6318	Anova	DT	0.718	RF	DT	0.652	Anova	DT	0.6211	Anova	Cos	0.5151
**64**	**RF**	**LR**	**0.9789**	**RFE**	**ET**	**0.979**	**RFE**	**KNN**	**0.8746**	**RFE**	**KNN**	**0.9911**	**RFE**	**LR**	**0.8379**
	RFE	LR	0.9777	RFE	LR	0.9782	RFE	LR	0.8688	RFE	LR	0.9892	ET	LR	0.8136
	RFE	Cos	0.977	RF	LR	0.9731	RFE	RF	0.8682	RF	LR	0.9862	RF	LR	0.8135
	RFE	ET	0.976	RFE	KNN	0.9727	RF	LR	0.8595	RFE	SVM	0.9843	RFE	SVM	0.7658
	RFE	RF	0.9757	ET	ET	0.9721	RF	Corr	0.8585	ET	LR	0.9837	RFE	ET	0.7475
	RF	RF	0.9755	RF	ET	0.9719	RFE	ET	0.8578	RFE	ET	0.9833	ET	SVM	0.7474
	ET	LR	0.9741	ET	LR	0.9706	RF	KNN	0.8574	RF	KNN	0.9791	RF	SVM	0.7471
	RF	ET	0.9737	Anova	ET	0.9695	RFE	SVM	0.8568	RF	ET	0.9781	RF	ET	0.7428
	RFE	SVM	0.9733	RFE	RF	0.9693	Anova	KNN	0.8564	RF	SVM	0.9778	ET	ET	0.7386
	ET	RF	0.9728	RFE	SVM	0.9688	Anova	LR	0.8557	RFE	RF	0.9774	RF	RF	0.7349
	RFE	Corr	0.9709	RF	SVM	0.9662	ET	LR	0.8539	ET	KNN	0.9766	ET	RF	0.7331
	ET	ET	0.9691	Anova	LR	0.9661	RFE	Corr	0.8537	ET	SVM	0.9763	RFE	KNN	0.73
	RFE	KNN	0.9656	ET	SVM	0.9656	ET	Corr	0.8536	ET	ET	0.9762	RFE	RF	0.7295
	RF	Cos	0.9647	ET	KNN	0.9635	ET	KNN	0.852	RF	RF	0.9748	RF	KNN	0.7276
	RF	Corr	0.9631	RF	KNN	0.9624	RFE	Cos	0.8492	ET	RF	0.971	ET	KNN	0.7225
	RF	SVM	0.9595	ET	RF	0.9613	ET	RF	0.8453	RFE	Corr	0.9673	RFE	Cos	0.7157
	ET	Cos	0.9585	Anova	RF	0.9609	RF	SVM	0.8448	RFE	Cos	0.9612	RFE	GB	0.7076
	ET	Corr	0.9576	RF	RF	0.9608	Anova	SVM	0.8426	ET	Corr	0.9233	RFE	Corr	0.6891
	RF	KNN	0.9545	Anova	SVM	0.9601	RF	RF	0.8424	RF	Corr	0.9227	ET	GB	0.6725
	ET	GB	0.9504	Anova	KNN	0.9581	RF	ET	0.8422	RF	Cos	0.9215	RF	GB	0.6711
	ET	SVM	0.9501	RFE	Cos	0.9167	ET	Cos	0.8407	ET	Cos	0.919	Anova	LR	0.6514
	RFE	GB	0.9481	RFE	Corr	0.9028	ET	SVM	0.8402	Anova	RF	0.8944	RF	Corr	0.6502
	ET	KNN	0.9468	ET	Cos	0.8968	Anova	RF	0.8398	Anova	ET	0.8787	ET	Corr	0.646
	RF	GB	0.9464	RF	Cos	0.8942	RF	Cos	0.8362	Anova	LR	0.8479	RF	Cos	0.6264
	Anova	LR	0.8924	Anova	Cos	0.8928	ET	ET	0.8357	Anova	SVM	0.8378	ET	Cos	0.6173
	Anova	SVM	0.8743	ET	Corr	0.8851	Anova	Cos	0.8344	RF	GB	0.7936	Anova	KNN	0.6033
	Anova	ET	0.8733	RF	Corr	0.8834	Anova	ET	0.8306	ET	GB	0.7794	Anova	SVM	0.6023
	Anova	RF	0.8705	Anova	Corr	0.8772	Anova	Corr	0.815	Anova	KNN	0.7786	Anova	RF	0.6015
	Anova	GB	0.8352	ET	GB	0.8135	RFE	GB	0.7897	RFE	GB	0.7253	Anova	GB	0.596
	Anova	Cos	0.8334	Anova	GB	0.8094	Anova	GB	0.777	Anova	GB	0.7214	Anova	ET	0.5932
	RFE	DT	0.8292	RF	GB	0.8085	ET	GB	0.7689	Anova	Cos	0.7201	RFE	DT	0.5803
	Anova	KNN	0.8273	RFE	GB	0.805	RF	GB	0.7496	Anova	Corr	0.7098	RF	DT	0.5762
	ET	DT	0.826	ET	DT	0.7203	RFE	DT	0.6553	RF	DT	0.6653	ET	DT	0.5708
	Anova	Corr	0.8226	RFE	DT	0.7196	Anova	DT	0.6526	ET	DT	0.6588	Anova	Corr	0.5675
	RF	DT	0.8173	RF	DT	0.7163	ET	DT	0.6498	RFE	DT	0.6502	Anova	DT	0.5466
	Anova	DT	0.6803	Anova	DT	0.7128	RF	DT	0.646	Anova	DT	0.6249	Anova	Cos	0.5222
**96**	**RF**	**LR**	**0.9808**	**RF**	**LR**	**0.979**	**RFE**	**LR**	**0.8697**	**RFE**	**KNN**	**0.9933**	**RFE**	**LR**	**0.847**
	RFE	LR	0.9787	RFE	LR	0.9779	RF	KNN	0.8657	RF	LR	0.9918	ET	LR	0.8292
	RF	RF	0.9774	ET	LR	0.9768	RF	LR	0.8643	RFE	LR	0.9916	RF	LR	0.8285
	RFE	Cos	0.977	RFE	ET	0.9765	ET	LR	0.8634	ET	LR	0.9909	RFE	SVM	0.7715
	RFE	RF	0.9762	ET	ET	0.9734	RFE	RF	0.8633	RF	SVM	0.9877	RF	SVM	0.7653
	ET	LR	0.9761	RF	ET	0.973	RF	Corr	0.863	RF	KNN	0.9877	ET	SVM	0.7602
	ET	RF	0.9758	Anova	LR	0.971	ET	Corr	0.8629	ET	KNN	0.987	RF	ET	0.7514
	RF	ET	0.9746	RF	SVM	0.9675	RFE	KNN	0.8628	RFE	SVM	0.9867	RFE	ET	0.7481
	RFE	ET	0.9744	ET	SVM	0.9673	ET	KNN	0.8613	RF	ET	0.9857	ET	ET	0.744
	RFE	Corr	0.9727	RFE	SVM	0.9672	Anova	KNN	0.8596	RFE	ET	0.9856	ET	RF	0.7401
	RFE	SVM	0.9723	Anova	ET	0.9669	Anova	LR	0.8573	ET	SVM	0.9852	RFE	KNN	0.7387
	ET	ET	0.9699	RFE	RF	0.9662	RFE	SVM	0.853	ET	ET	0.9831	RF	RF	0.737
	RF	Cos	0.966	ET	RF	0.9624	ET	RF	0.8483	RFE	RF	0.9824	RF	KNN	0.7345
	RF	Corr	0.9639	RFE	KNN	0.9623	RFE	Corr	0.8477	RF	RF	0.9822	RFE	RF	0.7336
	RFE	KNN	0.9632	RF	RF	0.961	RF	SVM	0.8474	RFE	Corr	0.9821	ET	KNN	0.7292
	RF	SVM	0.9618	Anova	SVM	0.9589	RFE	ET	0.847	ET	RF	0.9806	RFE	Cos	0.7264
	ET	Cos	0.9616	RF	KNN	0.9584	ET	SVM	0.8467	RFE	Cos	0.9777	RFE	GB	0.7064
	ET	Corr	0.9611	Anova	RF	0.9581	RF	RF	0.8457	RF	Corr	0.9503	RFE	Corr	0.7023
	RF	KNN	0.9561	ET	KNN	0.9574	ET	Cos	0.8451	ET	Corr	0.9488	RF	GB	0.6797
	ET	SVM	0.9545	Anova	KNN	0.9475	RFE	Cos	0.8425	RF	Cos	0.9481	ET	GB	0.6794
	ET	KNN	0.9487	RFE	Cos	0.9182	RF	ET	0.8403	ET	Cos	0.9476	RF	Corr	0.6725
	ET	GB	0.9463	RFE	Corr	0.9046	ET	ET	0.8397	Anova	RF	0.9107	Anova	LR	0.6693
	RF	GB	0.9434	ET	Cos	0.8971	Anova	RF	0.837	Anova	ET	0.8999	ET	Corr	0.6584
	RFE	GB	0.9425	RF	Cos	0.8965	Anova	Cos	0.8354	Anova	LR	0.883	RF	Cos	0.6487
	Anova	LR	0.9157	Anova	Cos	0.8845	RF	Cos	0.835	Anova	SVM	0.8657	ET	Cos	0.6374
	Anova	ET	0.8996	ET	Corr	0.8835	Anova	SVM	0.8342	Anova	KNN	0.8057	Anova	KNN	0.6148
	Anova	SVM	0.8991	RF	Corr	0.882	Anova	ET	0.8302	RF	GB	0.7923	Anova	SVM	0.6114
	Anova	RF	0.8984	Anova	Corr	0.8713	Anova	Corr	0.7962	ET	GB	0.7885	Anova	GB	0.6097
	Anova	KNN	0.8578	RFE	GB	0.8175	RFE	GB	0.7801	Anova	Cos	0.7496	Anova	RF	0.6093
	Anova	GB	0.8569	ET	GB	0.8065	Anova	GB	0.7684	Anova	Corr	0.738	Anova	ET	0.5989
	Anova	Cos	0.8473	RF	GB	0.8056	ET	GB	0.7613	RFE	GB	0.7334	Anova	Corr	0.5835
	Anova	Corr	0.836	Anova	GB	0.8	RF	GB	0.7462	Anova	GB	0.7271	RF	DT	0.5768
	RFE	DT	0.8329	RFE	DT	0.7067	Anova	DT	0.6554	RF	DT	0.667	RFE	DT	0.575
	ET	DT	0.8247	RF	DT	0.7049	ET	DT	0.6464	ET	DT	0.6564	ET	DT	0.5745
	RF	DT	0.8173	ET	DT	0.7038	RF	DT	0.6461	RFE	DT	0.6467	Anova	DT	0.5469
	Anova	DT	0.6934	Anova	DT	0.699	RFE	DT	0.6418	Anova	DT	0.6282	Anova	Cos	0.5244

Mean AUC per Model Grouped by Platform and Marker Set Size

Table 6: External Validation: Best performing models

Marker Set Size	Feature Selection	Classifier	AUC
8	RFE	Extra Trees	0.938 +/- 0.015
16	RFE	Logistic Regression	0.9676 +/- 0.112
32	RFE	Logistic Regression	0.9849 +/- 0.0055
64	RFE	Logistic Regression	0.9904 +/- 0.0036
96	RFE	Logistic Regression	0.9920 +/- 0.025

The top performing feature selection and classification algorithms selected by AUC in the external validation experiment.

Table 7: External Validation: NCI-60 to TCGA mapping

NCI-60 Cell Line	TCGA Cell lines
Central Nervous System	Glioblastoma multiforme (GBM)<br>Brain Lower Grade Glioma (LGG)
Lung	Lung adenocarcinoma (LUAD)<br>Lung squamous cell carcinoma (LUSC)
Colon	Colon adenocarcinoma (COAD)<br>Rectum adenocarcinoma (READ)
Ovarian	Serous Cystadenocarcinoma (OV)
Renal	Kidney renal clear cell carcinoma (KIRC)<br>Kidney renal papillary cell carcinoma (KIRP)

This table indicates which feature selection and classification algorithm was ranked highest by AUC during the model selection and training phase of the external validation experiment.

Table 8: Accuracy per Cancer Type Grouped by Marker Set Size

		NCI-60 Cell Lines							
**TCGA Samples**	**Marker Set Size**	**ME**	**LE**	**CO**	**CNS**	**RE**	**BR**	**OV**	**LC**
CNS	8	0.0	0.0	0.0	1.0	0.0	0.0	0.0	0.0
CO	8	0.0144	0.0048	0.1779	0.0769	0.0048	0.0192	0.4135	0.2885
LC	8	0.0291	0.0058	0.0058	0.3081	0.1977	0.0174	0.0	0.436
OV	8	0.4074	0.1852	0.0	0.0	0.0	0.0	0.0	0.4074
RE	8	0.0	0.0	0.0649	0.7013	0.0	0.0	0.1039	0.1299
CNS	16	0.0	0.0	0.0	0.8246	0.1754	0.0	0.0	0.0
CO	16	0.0	0.0	0.9856	0.0	0.0144	0.0	0.0	0.0
LC	16	0.0	0.0058	0.064	0.0523	0.1512	0.0407	0.0	0.686
OV	16	0.0	0.2222	0.037	0.0	0.0	0.0	0.037	0.7037
RE	16	0.013	0.026	0.0649	0.0519	0.7403	0.013	0.0649	0.026
CNS	32	0.0	0.0877	0.0	0.9123	0.0	0.0	0.0	0.0
CO	32	0.0	0.0	1.0	0.0	0.0	0.0	0.0	0.0
LC	32	0.0	0.0581	0.2151	0.1279	0.2791	0.0814	0.0	0.2384
OV	32	0.0	0.7037	0.1481	0.0	0.0	0.037	0.1111	0.0
RE	32	0.0	0.0779	0.0	0.013	0.8571	0.0	0.039	0.013
CNS	64	0.0	0.386	0.0	0.5965	0.0175	0.0	0.0	0.0
CO	64	0.0	0.0	1.0	0.0	0.0	0.0	0.0	0.0
LC	64	0.0	0.0872	0.064	0.0058	0.7035	0.0233	0.0	0.1163
OV	64	0.0	0.4074	0.1481	0.0	0.1111	0.0741	0.0741	0.1852
RE	64	0.013	0.0909	0.0	0.0	0.8961	0.0	0.0	0.0
CNS	96	0.0	0.4386	0.0	0.5351	0.0263	0.0	0.0	0.0
CO	96	0.0	0.0	1.0	0.0	0.0	0.0	0.0	0.0
LC	96	0.0	0.1802	0.2209	0.0174	0.5233	0.0465	0.0	0.0116
OV	96	0.0	0.4074	0.2222	0.0741	0.037	0.0	0.2593	0.0
RE	96	0.0	0.0	0.0	0.0	1.0	0.0	0.0	0.0

This table indicates the percentage of each TCGA cancer type that was classified to each NCI-60 cell line. The TCGA samples are grouped by marker set size. These predictions were made using the model specified in Supplemental Table 6.
